# High-definition neural visualization of rodent brain using micro-CT scanning and non-local-means processing

**DOI:** 10.1186/s12880-018-0280-6

**Published:** 2018-10-30

**Authors:** Ko-Chin Chen, Alon Arad, Zan-Ming Song, David Croaker

**Affiliations:** 1 The Canberra Hospital, Yamba Drive, Garran, ACT 2605 Australia; 2 Massachusetts Institute of Technology, Cambridge, MA 02139 USA; 3 Automated Analytics, Sugar Land, TX 77479 USA; 4 Medical School, Australian National University, Canberra, ACT 2601 Australia

**Keywords:** Micro-CT, Neuroimaging, NLM image processing

## Abstract

**Background:**

Micro-CT holds promising potential for phenotyping and histological purposes. However, few have clarified the difference in the neuroimaging quality between ex vivo and in vivo micro-CT scanners. In addition, no direct comparison has been made between micro-CT scans and standard microscopy. Furthermore, while the efficacy of various stains for yielding soft-tissue contrast in CT scans have been compared in other studies for embryos, staining protocols for larger samples have yet to be clarified. Lastly, post-acquisition processing for image enhancements have not been addressed.

**Methods:**

Comparisons of postnatal rat brain micro-CT scans obtained through custom-built ex vivo and commercially available in vivo micro-CT scanners were made. Subsequently, the scanned rat brains were then H&E stained for microscopy. Neuroanatomy on micro-CT scanning and 4× microscopy of rat brain were compared.

Diffusion and perfusion staining using iodine or PTA were trialled on adult and neonatal encapsulated rat brains. Different combinations of stain concentration and staining time were trialled.

Post-acquisition denoising with NLM filter was completed using a modern General-Purpose Graphic Processing Unit (GPGPU) and custom code for prompt processing.

**Results:**

Ex vivo micro-CT scans of iodine-stained postnatal rat brains yields 3D images with details comparable to 4× H&E light micrographs. Neural features shown on ex vivo micro-CT scans were significantly more distinctive than those on in vivo micro-CT scans.

Both ex vivo and in vivo micro-CT scans required diffusion staining through small craniotomy. Perfusion staining is ineffective. Iodine staining was more efficient than PTA in terms of time.

Consistently, enhancement made by NLM denoising on in vivo micro-CT images were more pronounced than that on ex vivo micro-CT scans due to their difference in image signal-to-noise indexes.

**Conclusions:**

Micro-CT scanning is a powerful and versatile visualization tool available for qualitative and potential quantitative anatomical analysis. Simple diffusion staining via craniotomy with 1.5% iodine is an effective and minimal structural-invasive method for both in vivo and ex vivo micro-CT scanning for studying the microscopic morphology of neonatal and adult rat brains. Post-acquisition NLM filtering is an effective enhancement technique for in vivo micro-CT brain scans.

**Electronic supplementary material:**

The online version of this article (10.1186/s12880-018-0280-6) contains supplementary material, which is available to authorized users.

## Background

Non-destructive whole-volumetric phenotyping studies through three-dimensional (3D) image reconstruction have gained increasing popularity among biomedical researchers over recent decades, especially with recent advancements in imaging acquisition and processing techniques. Three-dimensional visualization techniques may be divided into two categories: serial sectional image reconstruction and whole-volume imaging. The former includes confocal microscopy, episcopic microscopy [1], and the latter includes optical projection tomography (OPT) [2], micro-magnetic resonance imaging (micro-MRI) [3, 4], and micro-computed tomography (micro-CT) [5].

Whole-volumetric imaging is superior to serial imaging in many aspects. Serial imaging reconstructions such as confocal microscopy or confocal laser scanning microscopy (CLSM) can provide detailed and fluorescence-labelled representations of the sample, but of only limited thickness, typically <2–300 μm due to the need for optical transparency [6]. Although image reconstruction may be employed to obtain the entire 3D view of the sample, the process is laborious and often with operator-dependent results. Episcopic microscopy overcomes this shortfall by adopting automatic serial slice alignment, and reconstructs relatively well-preserved images; however, such a process destroys the tissue sample post-imaging and prohibits further sample use [1, 7]. Whole-volumetric imaging, on the other hand, does not have the above restrictions. For example, optical projection tomography (OPT) can be used to localize and measure structures within a whole organ, but only for restricted sample thicknesses [8]. Micro-MRI can offer great soft-tissue contrast with adequate resolution images, but its use is limited by its restricted availability, high cost and extended scanning time [4, 9, 10]. Micro-CT is now a widely accessible imaging modality, offering a mean for accurate visualization of three-dimensional structures, quantitative volumetric measurements, and tissue characterizations such as bone stress and vascular density [11–15]. Although many are still unaware of this versatile tool, micro-CT has gradually becoming a popular whole-volume scanning research method, in large part due to its versatility for volume exploration, ease of tissue preparation, and quantitative analysis potentials.

Although many recent papers have published various staining techniques for successful micro-CT scanning of biological samples, including chicken embryo, *Xenopus* embryo, mouse embryo, tumour angiogenesis, and other animal internal organs [5, 16, 17], few study have clarified the scanning protocols for micro-CT scans on postnatal model animals or made direct comparisons between the ex vivo micro-CT, in vivo micro-CT, and histology scans of rat neuroanatomy. Furthermore, even though prior study has suggested staining with iodine or phosphotungstic acid (PTA) were efficient and effective for embryos, no comments have been made on their staining power for postnatal animals [5]. In addition, the effect of iodine and PTA staining on subsequent microscopy processing has not been illustrated in the past. Lastly, we are also not aware of studies trialling image processing protocols on the rat brain micro-CT scans, which may facilitate future quantitative analysis.

Our study aims to complement previous studies by illustrating the following:

1. Simple, safe, and effective staining method for successful micro-CT scans of both small and large rat brains.
2. The potential neuroanatomical details of rat brain and image magnification and resolution achievable by micro-CT scanning using one of the most high-powered ex vivo micro-CT scanners available currently.
3. Validate neuroanatomical information of micro-CT scan by direct comparison with H&E light microscopy.
4. The difference in image quality between in vivo and ex vivo micro-CT scans of rat brain.
5. Effectively improve image clarity and potentiate organs of interest through non-local means (NLM) denoising algorithms.

## Methods

### Compliance with ethical practice

All tissues and animals used in this study were handled with strict adherence to the requirements of the ACT Health Human Research Ethics Committee (ACTH-HREC) and Australian National University Animal Experimentation Ethics Committee (ANU-AEEC), project number A2011/67.

### Differences between the principles of ex vivo and in vivo micro-CT setups

**Fig. 1:**
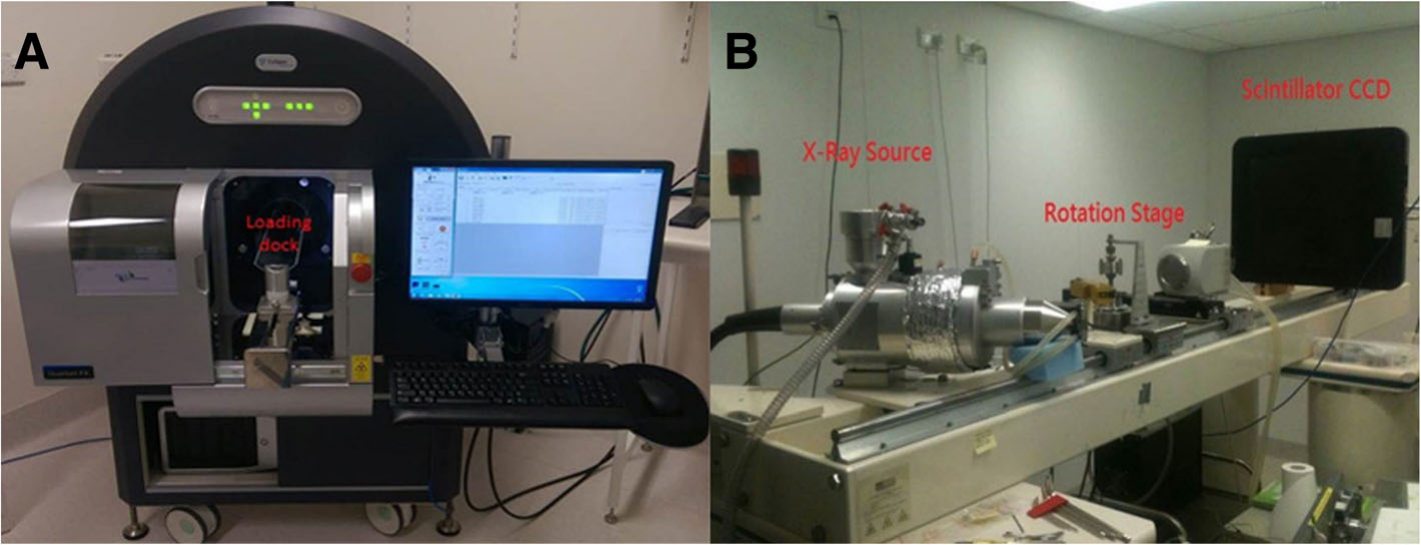
In vivo and ex vivo micro-CT machines utilized in this study. **a** The Caliper Quantum FX machine representing in vivo micro-CT setup, with loading dock labelled and situated in between the rotating X-ray source and detectors; X-ray source and detector are hidden within the casing. **b** The custom-built ex vivo micro-CT scanner by ANU applied mathematical department. The rotational sample stage is placed in-between the adjustable X-ray source and scintillator/detector for maximal magnification

We have chosen the Caliper Quantum FX machine (Fig. 1a) as representative of in vivo micro-CT setup and a custom-built micro-CT system by ANU Applied Mathematical Department as representative of ex vivo micro-CT setup (Fig. 1b).

The in vivo micro-CT system is set up as a mini-medical CT system. It incorporates an X-ray source and detectors rotating around a stationary sample, achieving a spatial resolution of 10–100 μm/voxel, which is restricted by the fixed distance between X-ray source and detector. In the Caliper Quantum FX, the tissue specimen was placed in a stationary loading dock positioned between the rotating system of X-ray source plus detector. The scanning time was pre-set between 17 s and 4.5 min, depending on the field of view (FOV; ranging from 5 to 75 mm in diameter) and imaging quality selected (ranging from “standard” to “fine”). The advantages of in vivo micro-CT scanners including a shorter scanning time and lower radiation exposure, both of which were essential for live animal study. The resultant images were stored as DICOM series and visualized with FIJI and Drishti, both of which were open-source software [18, 19].

In contrast, the ex vivo micro-CT system involves a specimen placed on a rotating stage between a stationary but adjustable system of X-ray source and detector. Its main focus is to achieve high spatial resolution, up to 1 μm/voxel, with high signal-to-noise ratio images. These benefits are achieved at the expense of longer scanning time and hence higher radiation dose. It is therefore mainly used for tissue study. The custom-built micro-CT system at the ANU Department of Applied Mathematics requires the sample to be fixed within an aluminium tube before being placed on the rotational sample stage between the adjustable X-ray source and scintillator-coupled-CCD, at a position that depends on the resolution desired. All samples were allocated at least 15 h of scanning time, with a subsequent 8 h of image-processing time required at the National Computational Infrastructure (NCI). The maximal achievable resolution is 1 μm/voxel, limited by the physical size of the sample. The resultant images were stored as netCDF files and visualized with Drishti. Segmentation for organs of interest was performed semi-automatically using Drishti paint, a component of Drishti open-source software [18].

As a side note, because the adjustable X-ray source can be brought much closer to the sample (limited by the sample size) with ex vivo than in vivo micro-CT setup, higher resolution is achievable. The same cannot be achieved with in vivo micro-CT setup due to current constructional challenges of fitting X-ray source and sufficient number of detectors onto a small rotational stand.

### Tissue samples and staining preparations for micro-CT scanning

#### Adult rats and neonatal rat culling

Five fully-grown rats (>21 and 27 days) and thirteen 24- and 48-h old rats were over-anaesthetized with 5% isoflurane and then culled via abdominal aortotomy. Each rat was weighed. One adult rat was prepared through the perfusion protocol and the remaining rats were prepared via the diffusion protocol, as described below.

#### Perfusion protocol for adult rat brains

To test the staining potential of perfusion protocols, we culled one 27-days-old rat via cardiotomy with a 22G cannula. The rat was then perfused with 200 mL of 0.1 M phosphate-buffered saline (PBS) solution and subsequently 200 mL of 4% formalin for successful fixation, indicated by tail rigidity; each stage took about 45 min. The perfusate existed the vascular system through inferior vena cava venotomy. Next, the rat was perfused with a series of progressively concentrated ethanol solutions (20%, 50%, 70%, 90%) over 1 h at each concentration to replace intravascular formalin, for subsequent staining. Staining was then attempted by perfusing the animal with 1.5% iodine (in 90% ethanol) through left ventricles for 2.5 h in a lab basin.

#### Diffusion staining protocols for encapsulated adult and neonatal rat brains and isolated rat brains

To explore the alternative staining techniques, we attempted diffusion staining by the following. Four adult rats’ heads (average age 25 days) were isolated from the neck and up following culling. A diamond-shaped craniotomy of 20-mm in diameter on parietal bones was created for staining. The craniotomy was completed firstly by a superficial cross-incision of 20 mm in diameter (through the thickness of the skull) using a dissection scalpel followed by diagonal excisions of skull using iris scissors. Superficial dissection was carefully performed to avoid brain tissue damage. Minimal damage of the brain was confirmed by the absence of brain tissue destruction in the following micro-CT scans.

Four-percent formalin was first used to fixate the rat head for 24 h followed by washout with graded ethanol in the following steps. Each fixed tissue was immersed in progressive concentrated ethanol (EtOH) series: 20, 50, 70, and 90% for 1 day each to replace formalin solution. Each sample was then stained in 1.5% iodine (with 90% EtOH) or 0.5% phosphotungstic acid (PTA) or 1% PTA in 70% EtOH for various durations (listed in Additional file 1: Table S1) as attempts to find the most effective staining time for successful CT scanning. Contrast concentration was decided based on the successful results of previous studies on embryo staining [5, 16].

Similarly, diffusion staining on 13 neonatal rat brains was achieved through similar processes: isolation of upper body from the level of diaphragm, creation of a 5-mm in diameter diamond-shaped craniotomy on parietal bones, 4% formalin-fixation for storage, progressive ethanol washout prior to staining, and diffusion staining with 1.5% iodine, 0.5% PTA, or 1.0% PTA for various periods (listed in Additional file 1: Table S1).

Lastly, three 24-h-old rat brains were dissected and fixed as described above prior to staining with 1.5% iodine and 1.5% PTA, respectively. Macroscopic views of PTA- and iodine-stained brains are shown by Additional file 2: Figure S1 and Additional file 3: Figure S2.

### Hematoxylin and eosin (H&E) histology preparations for comparison with micro-CT scans

To confirm the neuroanatomical details of the micro-CT scan and assess the potential tissue distortion created by the iodine or PTA staining process, as shown by the macroscopic brown-discoloration of brain tissues due to iodine staining (Additional file 2: Figure S1 and Additional file 3: Figure S2), we processed the stained rat samples with standard H&E protocols [20].

H&E processing were conducted in the following steps. The iodine- or PTA-stained tissues were first sectioned sagittally into blocks of 4 mm in thickness to fit in cassettes. These tissue-blocks were then transferred to 90% EtOH for 48 h for contrast washout and dehydration. Next, the alcohol was cleared by xylol prior to paraffin embedding at 60 °C. Following embedment, samples were sliced into tissue-sheets of 4 μm in thickness using a microtome. These tissue-sheets were then laid in water-bath of 5–6 °C to minimize wrinkles while being positioned onto labelled-glass slides. These slides were dried overnight at 37 °C.

H&E staining was completed by progressive staining. The slides were placed in alum-haematoxylin solutions until dark red colour was visualized. This was followed by washing and then ‘bluing’ with lithium carbonate solution. The slides were then washed before counter-stained with 0.5% eosin alcoholic solution.

All of the H&E slides were then examined with an Olympus IX71 Microscope with 4×, 20×, and 40× magnification.

### Post-acquisition image processing: non-local means (NLM) denoising

To improve the micro-CT image clarity, we denoised the acquired images to enhance image signal-to-noise ratios using NLM algorithm, which was first developed by Buades et al. [21]. The algorithm was based on the premises that similarity exists between different regions of an image as long as these region sizes were kept relatively small. Using these similarity characteristics, image noise could be removed through averaging the similar parts of the image. For more accurate removal of the image noise, we used standard NLM with locally adaptive estimates for both types of micro-CT scans. Traditionally, this process was time-consuming due to the high demand on computational power thus making its computer-processing-unit (CPU) runtime unacceptably high for all but the smaller 2-dimentional (2D) images. However, we adopted parallel processing using a General-Purpose Graphic Processing Unit (GPGPU) and shortened the processing time to 1-min and thirsty seconds for a 512^3^ sample, rendering this process effective and efficient for regular research use.

This code was implemented on an Intel (R) Core ™ i7-4770 K CPU @3.5GHz with 32G of RAM and Nvidia GeForce GTX Titan Black Kepler GK110 architecture running Linux. The processed CT data were then reviewed using Fiji [22].

#### Implementation of the NLM algorithm

A brief description of our NLM implementation is provided as follow. The vast majority of the NLM runtime is spent computing the sum of squared differences (ssd). The ssd computation for adjacent voxels is highly similar. We can exploit this similarity by using a pseudo moving average filter (MAF) to perform the ssd from one pixel/voxel to the next. For 2D, the square area of intensities used for one voxel’s ssd search neighbourhood differs from an adjacent voxel’s search neighbourhood only in the end rows or columns. The same principle applies in 3D, adjacent cubes differ only in two end slices.

In 3D, if M is the number of voxels of the inner ssd cube and N is the total number of cubes that we performed the ssd on, then the total number of difference operations: with no MAF, we performed N × M^3^ difference operations; with the MAF, we performed M^3^ + (N − 1) × 2 × M^2^ difference operations. For a large N, this becomes a significant reduction in computation. As a simple example, if M is 100 voxels and N is a billion, the second operation is then a two-orders of magnitude less operation.

Most implementations of the NLM require the user to specify at least three parameters: an estimate of the image noise standard deviation, an outer search window size, and an inner search neighbourhood size. All these factors influence the quality of the de-noise operation. An accurate estimate of the noise, the size of the search window and neighbourhood is generally impossible and often the user has not even the vaguest notion of what the optimized values may be. This leads to tedious and time-consuming testing and retesting of the de-noising operation with different parameters until acceptable results are obtained. Our implementation does not require these unknown and often arbitrarily chosen parameters to be specified. Our implementation automatically computes an estimate of the noise in the local area of the pixel/voxel being de-noised. Each local noise estimate is combined with internally computed parameters determined from various image properties such as the number of intensity values i.e. 256 or 65 K, to compute the final smoothing parameter of the NLM algorithm automatically.

The computation of the smoothing parameter and estimation of the image noise is adapted from Coupe et al. [23]. Our implementation differs from theirs in the computation of a unique noise estimate for local sub blocks of the image, as opposed to a single value for the entire image. We also consider the gray scale value (gsv) intensity range in the calculation of the smoothing parameter. The inclusion of the gsv intensity range was found empirically to be a significant factor in the success of the automatic de-noising over a range of different image types i.e. CT, scanning electron microscopy (SEM), ultrasound etc. Our implementation of NLM follows the original model of Additive Gaussian White Noise (AGWN) but also allows for some adjustment of shape of the Gaussian kernel to better approximate the real noise distribution for images with non-AGWN.

As with Coupe et al. [23], we also concluded that the best de-noising quality is achieved with a local estimate of the noise, the correction factor for non-AGWN images, and fixed window sizes. Little, if any, gain in quality was obtained with larger search window sizes. It is obvious that in many cases, image quality degrades with larger search window sizes as the statistics of the similarity between these regions degrades.

## Results

### Simple tissue preparation for successful micro-CT scanning

In the first part of study, we proceeded to elaborate the simple staining process for postnatal rat samples required to achieve successful micro-CT scanning of the brains. The results of various combinations of contrasting agent, stain concentration, and contrast types were summarized in Additional file 1: Tables S1.

#### Staining of encapsulated postnatal rat brains for successful micro-CT neural imaging

**Fig. 2:**
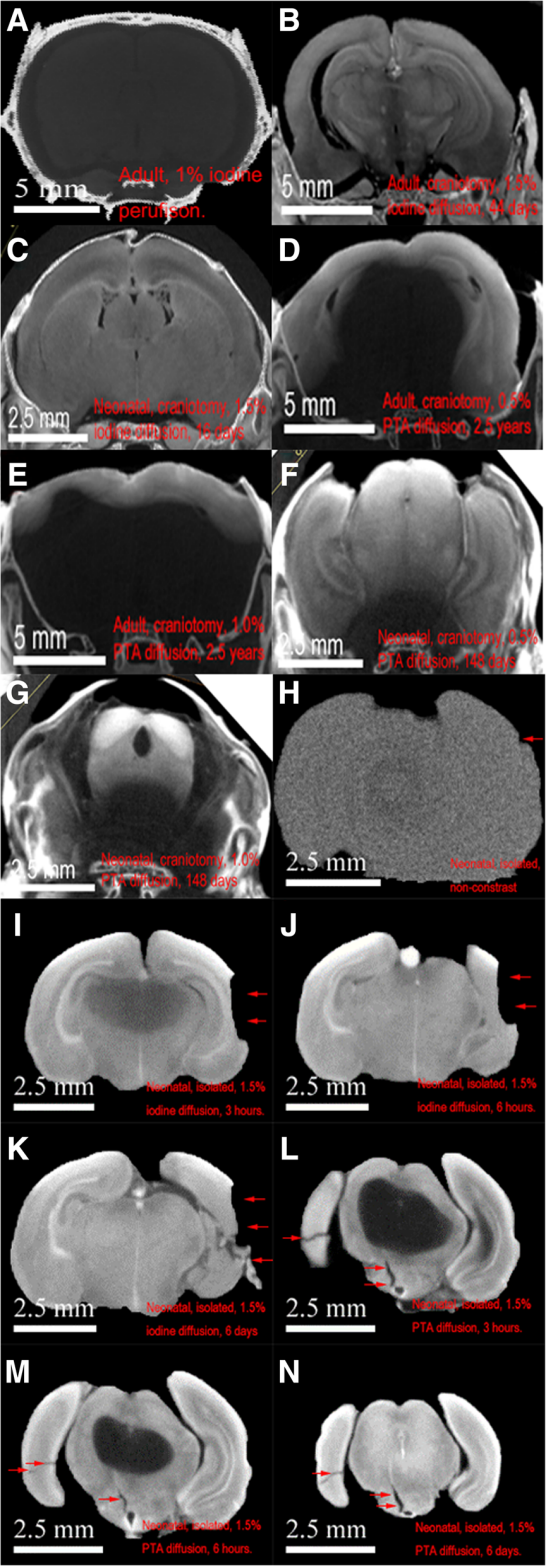
Successful tissue differentiation on micro-CT scanning demonstrate iodine diffusion staining technique is practical for both large (age of 25 days) and small (age of 36 h) postnatal encapsulated rat brain, Figure (**a**–**g**). (**a**) demonstrates unsuccessful neural-tissue scanning using 1.0% iodine perfusion staining for adult rat, whereas (**b**) and (**c**) show 1.5% iodine diffusion staining yielded good neural tissue contrast with adult and neonatal rat brains after 44 and 16 days, respectively. However, the same staining techniques using 0.5 and 1.0% PTA yielded little success on adult rat brains, (**d**) and (**e**), even after 2.5 years. Similarly, (**f**) and (**g**) show incomplete tissue differentiation of neonatal rat brains despite prolonged staining of 148 days using 0.5 and 1.0% PTA staining, respectively. Because PTA staining was non-uniform, selected coronal views were chosen to illustrate regions of incomplete tissue differentiation: (**a**–**d**) are anterior coronal views while (**e**–**g**) are posterior coronal views. Figure (**h**–**n**) illustrate iodine diffusion staining is equally effective but more efficient temporally to PTA staining for micro-CT scanning of small and isolated neural tissues. (**h**) shows lack of tissue contrast with no staining. (**i**–**k**) illustrate progressive improvement of tissue details on micro-CT scans with 1.5% iodine staining over time: 3, 6 h, and 6 days, respectively. Similarly, (**l**–**n**) show the same with 1.5% PTA staining over the same respective durations. Iodine staining was significantly faster

We first attempted 1.5% iodine perfusion staining on an adult rat of 27 days but with little success, as shown by Fig. 2a. Intracranial neural tissue exhibits little X-ray attenuation and minimal neuroanatomy was observed. The same rat’s head with intact skull was re-scanned following 14-days of 1.5% iodine diffusion-staining with no improvement in image quality (Figures not shown). Subsequently, 44-days of iodine-diffusion-staining through craniotomy was trialled with a 25-days-old rat’s head; micro-CT scan demonstrated differentiated intracranial details as shown by Fig. 2b: neural tissue differentiation became apparent. Consistently, same staining techniques also worked for neonatal rat brains but required only a staining period of 16 days; the resultant micro-CT scans yielded good neural anatomical information, as shown by Fig. 2c.

As an attempt to find an alternative contrast agent, we explored PTA diffusion staining using both 0.5 and 1.0% concentration solutions. Figure 2d and e are micro-CT scans of adult rat brains (25-days-old) derived from 2.5 years of PTA staining; both showed only peripheral brain of 2–4 mm in thickness. Similarly, as shown by Fig. 2f and g, incomplete neuroanatomy visualization was observed on micro-CT scans of neonatal rat’s head despite prolonged staining of 148 days. Interestingly, lower concentrations of PTA seemed to achieve more pervasive staining than higher, Fig. 2f vs g, although neither performed well.

#### Staining of dissected neonatal rat brains for successful micro-CT neural imaging

As the PTA staining was found to have suboptimal staining with encapsulated post-natal rat brain, we further evaluated its usefulness in staining small dissected rat brains, namely 1 cm^3^ in size. The result showed that PTA staining, although slow, did achieve full-staining and yield neural micro-CT scan quality similar to that derived from iodine staining. The comparison between the temporal series of iodine stained (Fig. 2i–k) and PTA stained (Fig. 2l–n) rat brains demonstrated the efficiency of iodine contrast. As Fig. 2j demonstrated, iodine staining was completed within 6 h; further staining did not improve image quality and tissue differentiation, Fig. 2k. On the other hand, only modest staining progress was made from 3-h PTA staining, Fig. 2l, and staining completion required 6 days, Fig. 2n. There were no significant image quality difference between the micro-CT scans derived from iodine-stained and PTA-stained rat brains.

In both cases, tissue differentiation as a result of staining was faster than when brain material was encapsulated in the skull, Fig. 2c. However, all scans revealed microscopic tissue damages of the rat brains indicated by arrows across Fig. 2h–n from dissection and handling despite macroscopically intact, as shown by Additional file 3: Figure S2. These brain damages were not seen on micro-CT scans of encapsulated brains, Fig. 2b and c.

### Image quality of micro-CT scans

In the second part of the study, we explored the image details and 3D-visualization capability of rat brain micro-CT scans.

#### High display versatility of micro-CT scan data using 3D-volume rendering

**Fig. 3:**
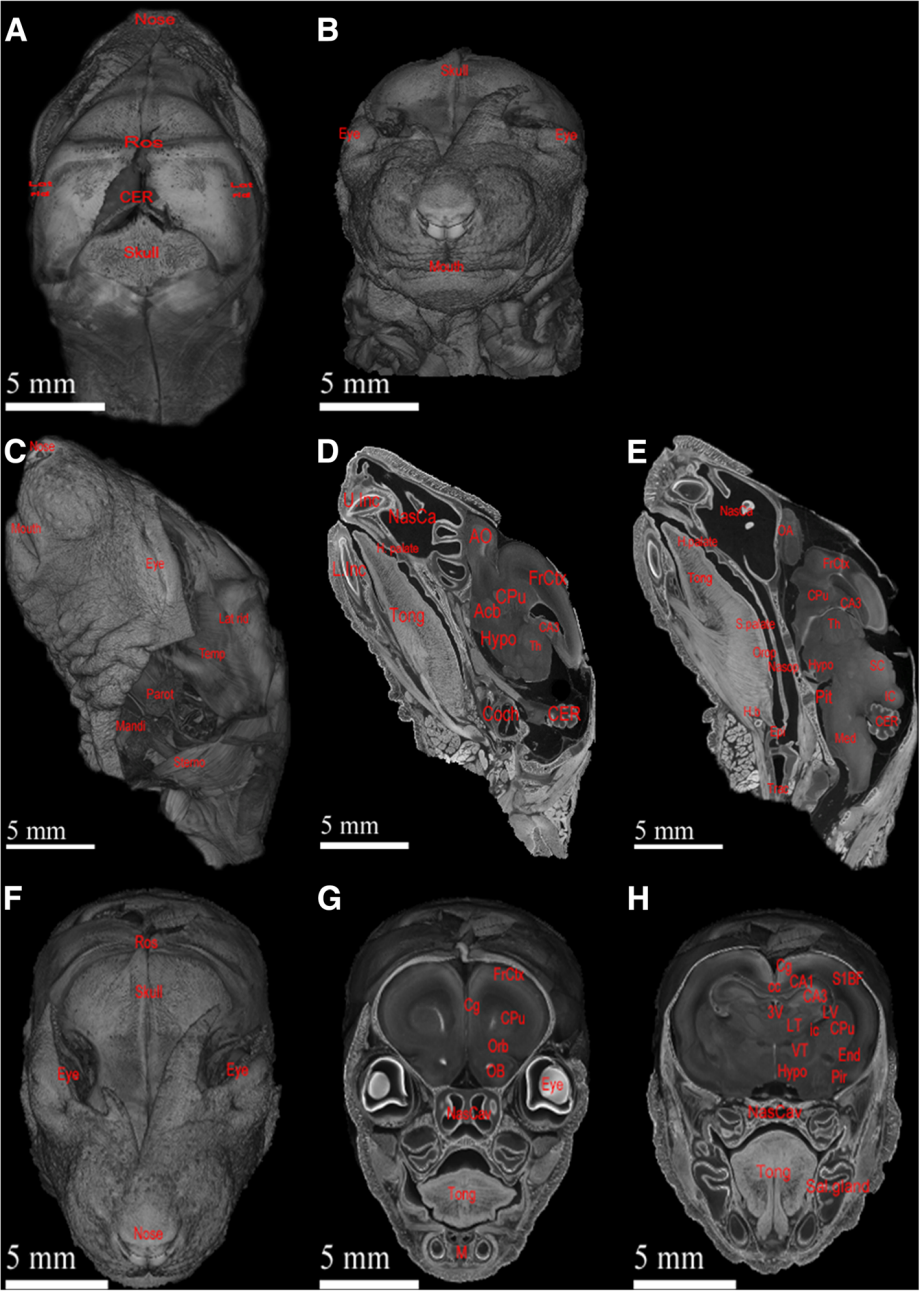
Volumetric rendering of ex vivo micro-CT scan of neonatal rat’s head enables detailed visualization and potential high-powered quantitative analysis. (**a** and **b**) illustrate respective anterior and posterior views of external features of rat’s head and neck, including mouth, nose, and muscle distributions. (**c**, **d**, and **e**) are respective external, parasagittal, and sagittal views of rat’s head and neck. (**f**, **g**, and **h**) are progressive coronal explorations of the same rat. These different views demonstrate the high-resolution power and flexibility of micro-CT scans. These exploration images show blood vessels, parotid glands, nasal anatomy, oral anatomy, and intracranial anatomy along with obvious muscular features throughout the head. Anatomical structures are labelled as follow: *Acb = accumbens nucleus; AO = anterior olfactory bulb; apons = anterior pons; ATh = anterior thalamus; CA1 = CA1 field of hippocampus; CA3 = CA3 field of hippocampus; cc = corpus callosum; CER = cerebellum; Cg = cingulate gyrus; Coch = cochlea; CPu = caudate putamen; End = Endopiriform nucleus; Epi = epiglottis; EPi = external plexiform layer; FrCtx = frontal cortex; H. b = hyoid bone; H. palate = hard palate; Hypo = hypothalamus; IC = inferior colliculus; ic = internal capsule; Lat rid = lateral ridge of skull; L. Inc = lower incisor; LT = lateral thalamus; LV = lateral ventricle; M = mandible; Mandi = mandibular gland; Med = medulla; NasCa = nasal cavity; Nasop = nasopharynx; OB = olfactory bulb; Orb = orbital cortex; OV = olfactory ventricle; Parot = parotid gland; Pir = piriform cortex; Pit = pituitary gland; PTh = posterior thalamus; ROS = rostral ridge of skull; Sal. Gland = salivary gland; SC = superior colliculus; S. palate = soft palate; S1BF = Somatosensory 1 Barrel Field; Sterno = sternomastoideus; Temp = temporalis; 3 V = 3rd ventricle; Th = thalamus; Tong = tongue; Trac = trachea; U. Inc = upper incisor; VT = ventral thalamus*

In this study, we demonstrated the investigatory power of micro-CT scanning by providing examples of the full visualization capability of current ex vivo micro-CT scanning through 3D-volume rendering. Figure 3s show the 3D-volume rendering of neonatal rat brain in high definition, 2048*2048*2048 voxels, with a resolution of 10.7 μm/voxel. Through manipulations of visual planes, one can identify and analyse organs of interest. For instance, the dorsal and ventral external features of the neonatal rats are illustrated in Fig. 3a and b, respectively. In addition, Fig. 3c–e show internal organs visualized in parasagittal and sagittal views, ranging from salivary glands, musculatures, nasal cavity, oral cavity, and neuroanatomy. Figure 3c and d show high-definition progressive views of the brain and identified organs such as olfactory bulb, cerebral cortex, cerebellum, and cochlea in structural-preserved manner. Similarly, progressive anterior to posterior axial views, Fig. 3f–h, provide alternative visualizations of cross-sectional rat brain anatomy that are difficult to appreciate in other views, such as cingulate gyrus, lateral thalamus and ventral thalamus. Overall, Fig. 3a–h demonstrate that through different visual-planes exploration, various external and internal features of buccal, nasal, and intracranial organs can be visualized with excellent tissue contrast. Additionally, simple quantitative size measurement can be made with reference to the scale bar listed.

#### Validating neuroanatomical information offered by micro-CT scans with H&E histology

**Fig. 4:**
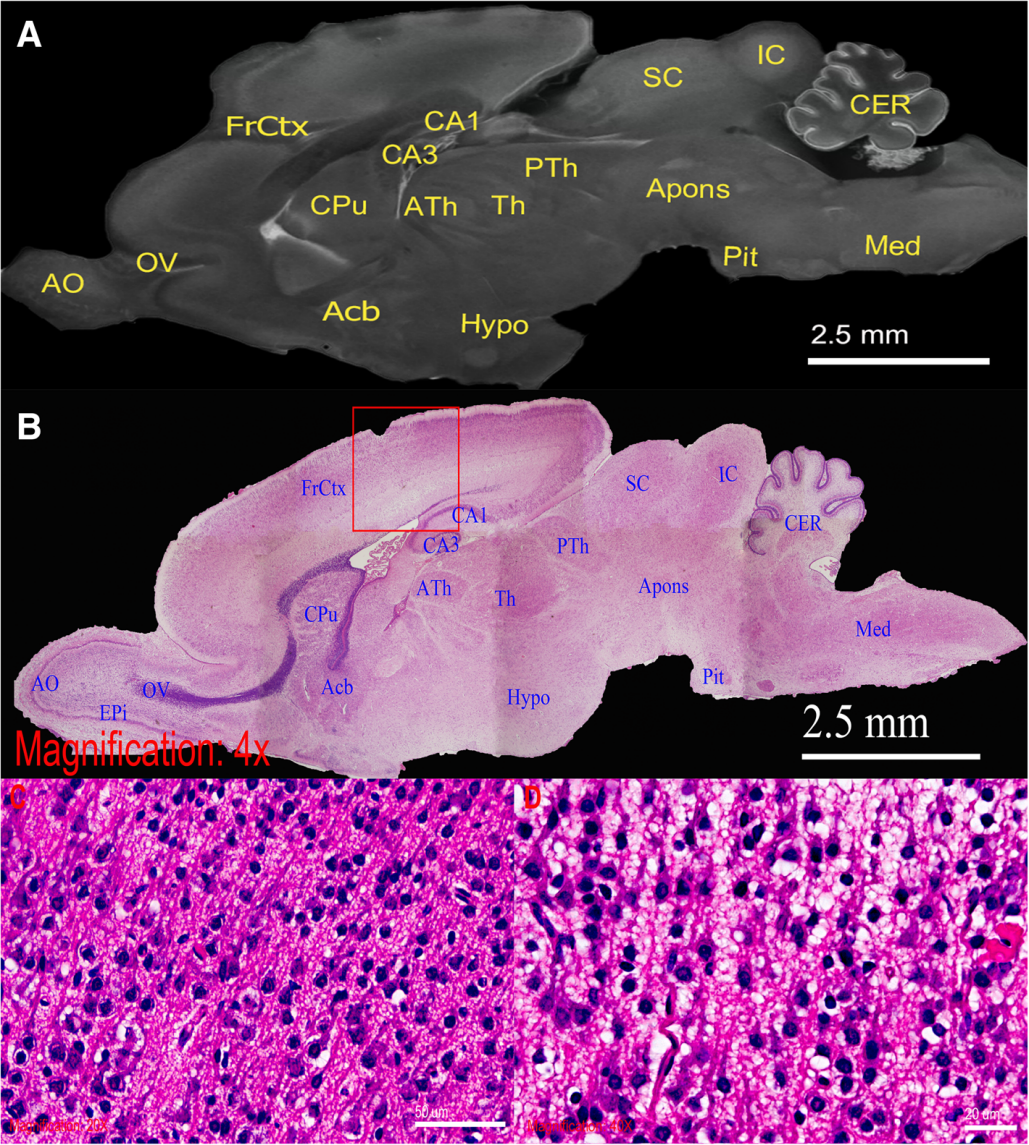
High-powered demonstration of rat brain by ex vivo micro-CT scan. Direct comparison between the sagittal section of rat brain in (**a**), micro-CT slice, and (**b**), H&E stained 4× light micrograph. Similar structural details are seen in these two imaging. (**c** and **d**) are respective 20× and 40× light micrographs of selected FrCtX region, demonstrating neuronal cell bodies. Anatomical structures of comparable visibility are labelled as follows: *Acb = accumbens nucleus; AO = anterior olfactory bulb; apons = anterior pons; ATh = anterior thalamus; CA1 = CA1 field of hippocampus;* CA3 = CA3 field of hippocampus; *CER = cerebellum; CPu = caudate putamen; EPi = external plexiform layer; FrCtx = frontal cortex; Hypo = hypothalamus; IC = inferior colliculus; Med = medulla; OV = olfactory ventricle; Pit = pituitary gland*; PTh = posterior thalamus; *SC = superior colliculus; Th = thalamus*

To validate micro-CT scanning method and explore potential microscopic tissue distortion as a result of the staining preparations for micro-CT scanning, we compared the ex vivo micro-CT scan, Fig. 4a, to H&E microscopy, Fig. 4b, both of which derived from the same iodine-stained rat brain. This comparison demonstrates that 3D rendering of the rat brain micro-CT scans offered neuroanatomical information comparable to that shown in 4× H&E microscopy. The grayscale distribution of micro-CT scans correlated strongly to the H&E distribution and therefore enabled successful neural structure distinguishment including olfactory bulb, caudate putamen, frontal cortex, thalamus, medulla, and cerebellum. Furthermore, the tissue integrity was well preserved in micro-CT scans when comparing to H&E light microscopy, Fig. 4b–d, which demonstrated micro-tears as a result of sectioning and H&E staining process. Moreover, successful light microscopy of iodine-stained rat brain demonstrated macroscopic discoloration by iodine, Additional file 2: Figure S1B, did not prohibit tissue from histology processing and higher magnifications of microscopy can still be achieved, Fig. 4c (20× magnification) and Fig. 4d (40× magnification), where nuclei can be visualized clearly.

#### Direct comparison of image quality of in vivo and ex vivo micro-CT scanning

**Fig. 5:**
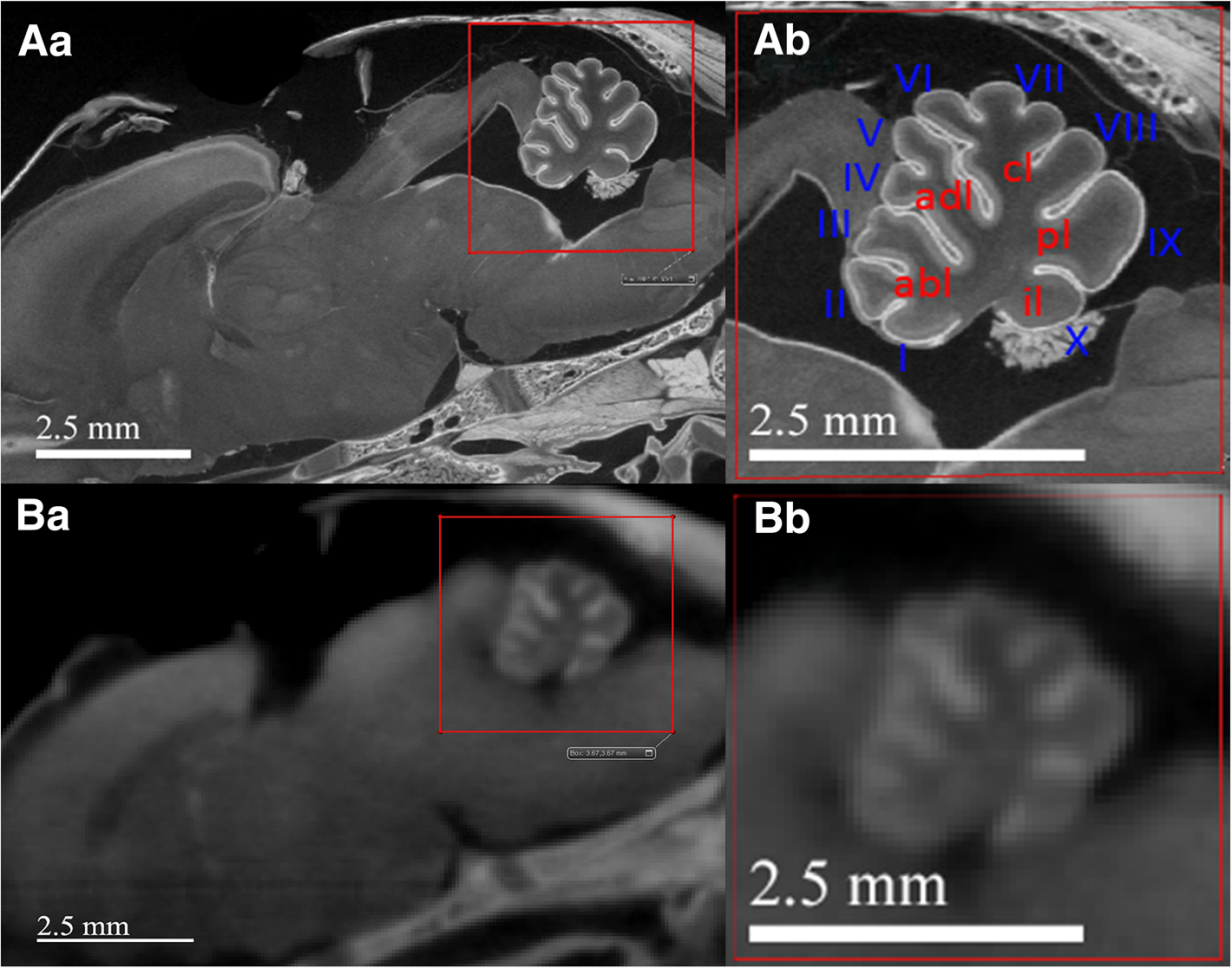
Direct comparison of ex vivo (spatial resolution of 10.7 μm/voxel) and in vivo (spatial resolution of 20 μm/voxel) micro-CT scans of the same 24-h-old rat’s head shows the image-quality difference between the two. (**Aa**) and (**Ba**) are sagittal illustrations of ex vivo and in vivo micro-CT scans, respectively. Gross neuroanatomy can be visualized in both scans with subtle difference appreciated by close-viewing, (**Ab**) and (**Bb**). Detailed cerebellar fissures, vermis (I–X), and lobes, can only be appreciated on ex vivo scans: *abl = anterobasal lobe; adl = anterodorsal lobe; cl = central lobe; pl = posterior lobe; il = inferior lobe*

We illustrated the image difference between the in vivo and ex vivo micro-CT scans of the same iodine-stained rat-brain through direct comparison, as shown by Fig. 5. This comparison demonstrated the in vivo micro-CT scan’s imaging limitations.

Both types of scans offered gross anatomical information; however, ex vivo micro-CT scans provided significantly more detailed neuroanatomy and sharper outlines than those of in vivo micro-CT scans. By comparing Fig. 5Aa and Ba, the respective ex vivo and in vivo micro-CT scans of a 24-h-old rat brain, one can clearly appreciate the frontal cortex, caudate putamen, superior and inferior colliculus, and trabeculae of the skull; all of which are well-defined in Fig. 5Aa. Higher magnification of the distinctive morphology in and around the cerebellum was chosen to further demonstrate how this difference can impact potential qualitative and quantitative analysis, as shown by Fig. 5Ab (ex vivo micro-CT scan) and Fig. 5Bb (in vivo micro-CT scan). While the rough outline of cerebellar fissures and vermis are still discernible in the in vivo micro-CT scan (Fig. 5Bb), intrinsic noise and oversaturation of the scan render boundaries much less crisply resolved than in the ex vivo micro-CT scan (Fig. 5Ab). Hence, one can easily define the ten cerebellar vermis and their respective sizes in Fig. 5Ab but not in Fig. 5Bb. This demonstrated the analytic limitations of in vivo micro-CT scans. In addition, volumetric measurements of brain based on segmentations of in vivo micro-CT scans differs slightly to that of ex vivo micro-CT scans, 424.75 mm^3^ vs 395.54 mm^3^. Although small, this discrepancy is due to the image quality difference resulting less precise segmentation from in vivo micro-CT scans.

### Post-acquisition image processing: non-local means (NLM) denoising

**Fig. 6:**
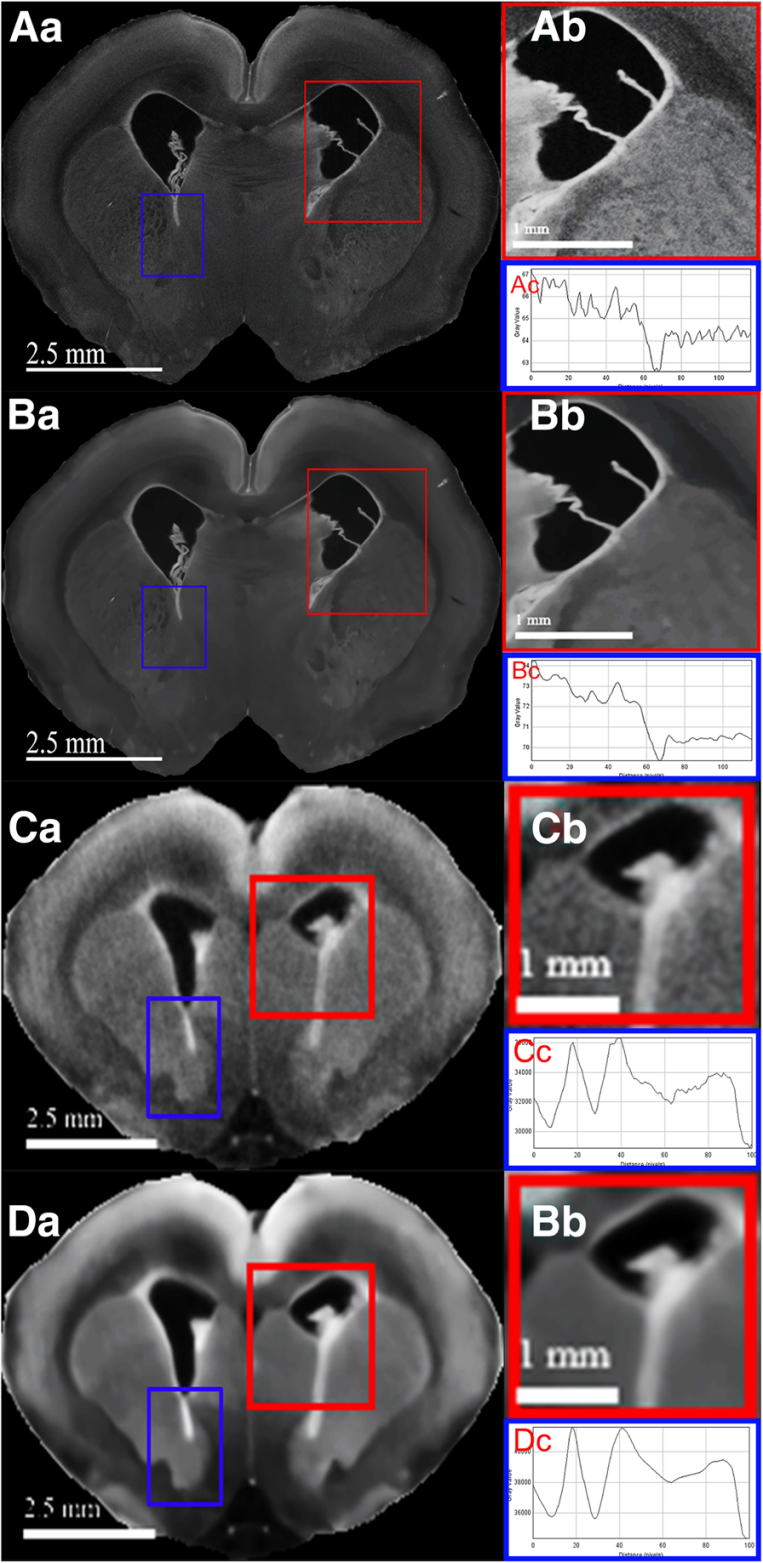
Neuroanatomical differentiation of both ex vivo and in vivo micro-CT scan are enhanced by NLM denoising. The original (**Aa**) and denoised (**Ba**) ex vivo scans illustrate sharper periventricular edges seen in (**Ba**) while gross neuroanatomical details are preserved in both. The difference in image noises is more obvious when comparing the respective magnified views, (**Ab**) and (**Bb**). Similar but more prominent edge enhancements is shown by the comparison of (**Ca**) and (**Da**), original and denoised in vivo micro-CT scans, respectively. The markedly improved signal-to-noise ratio is easily appreciated in the magnified denoised (**Db**) from the original (**Cb**) views. The respective grayscale line profile of each scan revealed reductions in intensity variability in denoised group, (**Bc**) and (**Dc**), in comparison to the originals, (**Ac**) and (**Cc**)

Post-acquisition image processing using NLM algorithm was incorporated into this study as attempts to improve CT image quality for better visualization of neural differentiation through denoising, which is particularly useful for in vivo micro-CT scans due to the high intrinsic images noise. Pre- and post-processing of ex vivo micro-CT scans, Fig. 6Aa and Ba, show organ boundaries are subtly enhanced by NLM processing. This is more evident in their respective magnified views, namely the bordering of caudate putamen and external capsule, as demonstrated by Fig. 6Ab and Bb. In addition, their respective grayscale line profiles illustrate reductions in intensity spread, Fig. 6Ac and Bc, demonstrating noise reduction following processing. Similarly, the same but more prominent effect can be seen from the processing of in vivo micro-CT scans, as shown by Fig. 6Ca and Da, demonstrating edge enhancements of caudate putamen, lateral ventricles, and external capsules; these difference are obvious in their respective magnified views, Fig. 6Cb and Db. The comparison of their respective grayscale line profiles also demonstrated decreases in intensity variability, Fig. 6Cc and Dc.

## Discussion

Through proper micro-CT setup, sample preparation, and image processing, informative neural micro-CT scans can be generated with postnatal animals. These scans enable internal visualization and potential quantitative analyses including volumetric and dimensional measurements by providing images with neural-tissue differentiation comparable, if not better, to micro-MRI brain scans, as illustrated Denic et al. [24]. Furthermore, micro-CT scans has a maximal spatial resolution of 1 μm/voxel with 16-h scanning time, significantly higher than 20 μm/voxel of micro-MRI achieved by 24 h of high-magnetic-fields scanning [4, 25]. Consistently, this high-magnification power of micro-CT provides anatomical and histological details comparable to those of 4× H&E light-microscopy, Fig. 4b. As a result of these characteristics, 3D rendering of the ex vivo micro-CT scans can show detailed and refined micro-neuroanatomy, Fig. 3. This function can therefore be applied to morphological study on genetic-disease model animals or cancer studies. Moreover, because tissue sample can be preserved following micro-CT scanning for H&E processing, Fig. 4c and d, micro-CT scan can serve as a screening tool for area of interest in the brain for further histology analysis. Although high-resolution micro-CT has the drawback of large dataset size, >4.6 GB/dataset, the high demand in computational and storage power for image analysis are met by the recent advancement in computing hardware, rendering this a practical research modality.

Although the difference between ex vivo and in vivo micro-CT has been addressed in previous studies, few have made direct comparisons of the quality difference of postnatal neural scans using these two modalities [26, 27]. In this study, we demonstrated the difference in magnification and resolution potentials of micro-CT scans using a custom-built ex vivo micro-CT scanner versus a commercial in vivo micro-CT scanner, as shown by Fig. 5. Consistent with expectations, our results have shown that ex vivo micro-CT scans offer finer detailed scans than in vivo micro-CT scans, due to the higher magnification achieved by having the X-ray source closer to the sample (i.e., a short source-sample distance, *SSD*). In theory, reducing the *SSD* increases the image noises and blurs edges, an effect clearly shown in Fig. 5Ba and Bb. However, image degradation can be minimized by using a low source current and careful focusing to minimize source spot size to obtain a best resolution of <2–3 μm/voxel while extending the scanning time to over 15 h to achieve high signal-to-noise ratio images. In comparison, commercial in vivo micro-CT scanners have limited magnifications and thus lower resolution due to the preset values of SSD. Furthermore, higher image noise in in vivo micro-CT scans is due to the shorter scanning time required to minimize the radiation exposure in live-animal scanning. In general, the best image quality needs the longest possible scanning time in either ex or in vivo micro-CT setups.

Based on the prior success of embryo study, we compared two contrast agents, iodine and PTA, for postnatal neural tissue staining [16]. The contrasts were chosen for their safety, ease of handling, and the likely effectiveness based on prior studies. Perfusion was first trialled in the hope of preserving rat skull integrity. Unfortunately, iodine staining was ineffective when introduced this way, likely due to poor penetration through the blood brain barrier and slow diffusion rate through capillary walls to end-organs [28, 29]. Contrary to prior embryo studies, simple diffusion staining with intact skull was also trialled with little success, suggesting contrast penetration through calcified skull was not possible [16]. Hence, diffusion-staining through a small craniotomy was subsequently adopted and found effective using iodine staining on all tested tissues. However, PTA was less effective as a stain using the same protocols on partially encapsulated tissues, especially on large adult rat brains; staining remained incomplete after 2.5 years, Fig. 2d and e, rendering this method impractical.

Due to concerns of prolonged staining may cause potential structural damage and prohibit tissues from future histology uses, as shown in Additional file 2: Figure S1, we tested PTA and iodine staining on dissected rat brains of small sizes with hope, Additional file 3: Figure S2. The result showed both stains yield similar quality of rat brain micro-CT images, as demonstrated by Fig. 2k and n. However, iodine was able to complete tissue staining faster than PTA due to its much higher penetration. In addition, iodine was better in staining poorly vascularized tissues such as the central nervous system, where PTA had little success in staining tissues with size greater than 1 cm^3^. This is likely due to the size and polarity difference of the two contrast agents: iodine is a non-polar molecule that is about 20 times smaller than the polytungstate anion of PTA [30–33]. Nevertheless, we found staining times to be significantly longer than previously described [5, 16]. To compensate for the lower penetration of PTA, limiting tissue volume to 1 cm^3^ and complete organ isolation through dissection may be required for successful staining. While these modifications to the method have proven effective, adopting such an approach defeats the purpose of using micro-CT, since dissection and excessive handling increase the risks of structural damage, even by experienced dissectors, as illustrated by the micro-tears shown by micro-CT scans, Fig. 2h through n. Based on our findings and the known difference in the properties of iodine and PTA, we recommend diffusion-iodine staining with partial craniotomy as the method of choice for micro-CT scans of postnatal rat brains. Additionally, successful H&E processing on both iodine- and PTA-stained brain tissues confirmed that prolonged contrast staining for micro-CT scanning compromises little, if any, tissue integrity and neuronal cell bodies can be visualized in higher magnification light micrographs, Fig. 4c and d. This suggests micro-CT scans may serve as a targeting tool for regions of interest and reduce unnecessary histopathology processing in pathology study, thus reduce labour and time.

Post-acquisition image processing through NLM filtering was performed to enhance image clarity and structural boundaries. NLM algorithm was chosen for its property of spatial detail preservation [21]. Although denoising only modestly improved ex vivo micro-CT scans, Fig. 6Aa and Ba, due to the scan’s high intrinsic signal-to-noise ratio, it has proven useful for in vivo micro-CT scans, which has high intrinsic image-noises, Fig. 6Ca. This image-noise was significantly reduced after processing, Fig. 6Da. These improvements are obvious in selected magnified views of caudate putamen, Fig. 6Cb and Db. We appreciate that commercially available in vivo micro-CT scanners are more widely available than custom ex vivo micro-CT scanners. By demonstrating the quality of in vivo micro-CT scans can be effectively improved through NLM algorithms, we support in vivo micro-CT data can also offer anatomical visualizations and accurate quantitative analysis. Lastly, denoising reduces the image noise, as demonstrated by the reduction in the variability of intensity profiles, Fig. 6Bc and Dc; this is an important step for the future developments of accurate automated segmentation for organs of interests.

## Conclusions

Micro-CT scanning, using either ex vivo or in vivo micro-CT scanners, is a powerful and effective neuroanatomy visualization modality for lab animals. Furthermore, micro-CT digital data is easy to store and manipulate. The anatomical details and accuracy offered by micro-CT scanning have been validated with the traditional H&E stained light micrograph. Moreover, the safety, simplicity, and tissue-preserving characteristic of the iodine-diffusion staining through craniotomy render micro-CT scanning a very easy-to-use study modality. Lastly, we appreciate that in vivo micro-CT scanners are more widely accessible and have a faster image processing time than ex vivo micro-CT scanners, the low signal-to-noise ratio due to the physical data acquisition restraints of in vivo micro-CT scans has been addressed. The improvement made by simple and efficient post-acquisition NLM processing reduces image degradation and enhances anatomical clarity, particularly with in vivo micro-CT scans, thus promoting versatile structural segmentation for quantitative analysis of animal morphology, including volumetric measurements. Based on described imaging methods, future studies may include: 1. internal organ visualization and quantitative analysis on animal models of genetic disease, e.g. Hirschsprung’s disease; 2. cancer treatment response studies on animal models; 3. identifying areas of interest for histological processing in pathology studies.

## Additional files

Additional file 1: **Table S1.** Summary of various tissue staining and micro-CT setups for different rat brains with respective image results. (DOCX 24 kb)

Additional file 2: **Figure S1.** Rat pup head after the removal of fur and creation of rhomb-shaped opening in skull for staining. (a) represents sample post-PTA staining and (b) post-iodine staining. (TIF 1074 kb)

Additional file 3: **Figure S2.** Stained isolated rat brain. (a) shows the effect of PTA staining and (b) iodine staining. (TIF 316 kb)

## Abbreviations

3V: 3rd ventricle
ACTH-HREC: ACT Health Human Research Ethics Committee
AGWN: Additive Gaussian White Noise
ANU-AEEC: Australian National University Animal Experimentation Ethics Committee
CA1: CA1 field of hippocampus
CA3: CA3 field of hippocampus;
cc: corpus callosum
CCD: X-ray coupled-charged device.
Cg: Cingulate gyrus
CLSM: Confocal laser scanning microscopy
Coch: Cochlea
DICOM: Digital imaging and communications in medicine
DSD: Detector-sample distance
End: Endopiriform nucleus
Epi: Epiglottis
EtOH: Ethanol
FOV: Field of view
gsv: gray scale value
H&E: Hematoxylin and Eosin stain
H. b: Hyoid bone
H. palate: Hard palate
ic: internal capsule
L. Inc.: Lower incisor
Lat rid: Lateral ridge of skull
LT: Lateral thalamus
LV: Lateral ventricle
M: Mandible
MAF: Moving average filter
Mandi: Mandibular gland
Micro-CT: Micro-computed tomography
Micro-MRI: Micro-magnetic resonance imaging
NasCa: Nasal cavity
Nasop: Nasopharynx
NCI: National Computational Infrastructure
NetCDF: Network Common Data Form
NLM: Non-local-means processing
OB: Olfactory bulb
OPT: Optical projection tomography
Orb: Orbital cortex
Parot: Parotid gland
PBS: Phosphate-buffered saline
Pir: Piriform cortex
Pit: Pituitary gland
PS: Pixel size
PSNR: Peak signal to noise ratio
PTA: Phosphotungstic acid
ROS: Rostral ridge of skull
S. palate: Soft palate
S1BF: Somatosensory 1 Barrel Field
Sal. Gland: Salivary gland
SDD: Source-detector distance
SEM: Scanning electron microscopy
SSD: Sample-source distance
ssd: sum of squared differences
Sterno: Sternomastoideus
Temp: Temporalis
Tong: Tongue
Trac: Trachea
U. Inc.: Upper incisor
VT: Ventral thalamus
