# Supplementary material for: High-definition neural visualization of rodent brain using micro-CT scanning and non-local-means processing
Source: BMC Med Imaging. 2018 Oct 30;18:38. doi: 10.1186/s12880-018-0280-6 (PMC6208172; doi:10.1186/s12880-018-0280-6)
Supplement: Supplementary file 1 — Table S1. Summary of various tissue staining and micro-CT setups for different rat brains with respective image results. (DOCX 24 kb) [file 12880_2018_280_MOESM1_ESM.docx]

| Tissue Type | Stain type | Stain concentration | Staining  method | Staining  duration | Micro-CT type | Results:  Excellent-Detailed anatomy  Good-Gross anatomy.  Poor-Gross anatomy, blurry  border.  Incomplete-Undifferentiated  anatomy |
| --- | --- | --- | --- | --- | --- | --- |
| Adult rat brain 1 | Iodine | 1.0% | Perfusion | 2.5 hours | *Ex vivo* | **Incomplete** - Figure 2A |
| Adult rat brain 2. | Iodine | 1.5% | Diffusion | 44 days | *Ex vivo* | **Excellent** - Figure 2B. |
| Adult rat brain 3 | PTA | 0.5% | Diffusion | 2.5 years | *In vivo* | **Incomplete** - Figure 2D |
| Adult rat brain 4 | PTA | 1.0% | Diffusion | 2.5 years | *In vivo* | **Incomplete** - Figure 2E. |
| Neonatal rat brain 1 | Iodine | 1.5% | Diffusion | 16 days | *Ex vivo* | **Excellent** - Figure 2C. |
| Neonatal rat brain 2 | PTA | 0.5% | Diffusion | 148 days | *In vivo* | **Incomplete** - Figure 2F |
| Neonatal rat brain 3 | PTA | 1.0% | Diffusion | 148 days | *In vivo* | **Incomplete** - Figure 2G |
| Isolated neonatal Rat Brain 1 | Non-Contrast |  |  |  | *In vivo* | **Incomplete** - Figure 2H |
| Isolated neonatal Rat Brain 2 | Iodine | 1.5% | Diffusion | 3 hours | *In vivo* | **Poor** - Figure 2I |
| Isolated neonatal Rat Brain 3 | Iodine | 1.5% | Diffusion | 6 hours | *In vivo* | **Good** - Figure 2J |
| Isolated neonatal Rat Brain 4 | Iodine | 1.5% | Diffusion | 6 days | *In vivo* | **Good** - Figure 2K |
| Isolated neonatal Rat Brain 5 | PTA | 1.5% | Diffusion | 3 hours | *In vivo* | **Incomplete** - Figure 2L |
| Isolated neonatal Rat Brain 6 | PTA | 1.5% | Diffusion | 6 hours | *In vivo* | **Incomplete** - Figure 2M |
| Isolated neonatal Rat Brain 7 | PTA | 1.5% | Diffusion | 6 days | *In vivo* | **Good** - Figure 2N |

**Table S1:** Summary of various tissue staining and micro-CT setups for different rat brains with respective image results.
