# Supplementary figures and images for: High-definition neural visualization of rodent brain using micro-CT scanning and non-local-means processing
Source: BMC Med Imaging. 2018 Oct 30;18:38. doi: 10.1186/s12880-018-0280-6 (PMC6208172; doi:10.1186/s12880-018-0280-6)

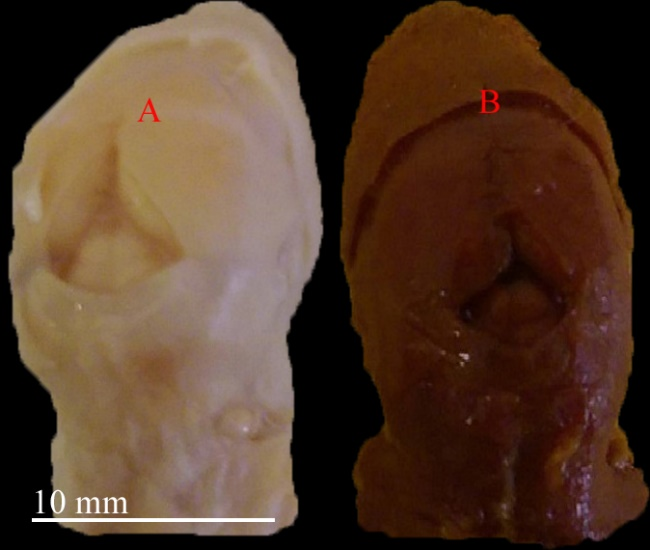

Supplement: Supplementary file 2 — Figure S1. Rat pup head after the removal of fur and creation of rhomb-shaped opening in skull for staining. (a) represents sample post-PTA staining and (b) post-iodine staining. (TIF 1074 kb) [file 12880_2018_280_MOESM2_ESM.tif]

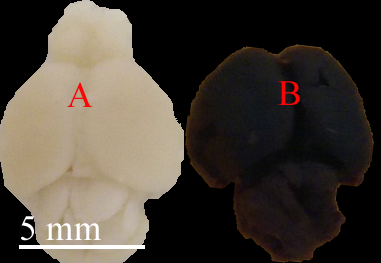

Supplement: Supplementary file 3 — Figure S2. Stained isolated rat brain. (a) shows the effect of PTA staining and (b) iodine staining. (TIF 316 kb) [file 12880_2018_280_MOESM3_ESM.tif]
